# Supplementary material for: Phenolic Composition of Crataegus monogyna Jacq. Extract and Its Anti-Inflammatory, Hepatoprotective, and Antileukemia Effects
Source: Pharmaceuticals (Basel). 2024 Jun 15;17(6):786. doi: 10.3390/ph17060786 (PMC11207108; doi:10.3390/ph17060786)
Supplement: Supplementary file 1 [file pharmaceuticals-17-00786-s001.zip › pharmaceuticals-3017416-supplementary.pdf]

MOLECULE	M/Z
Syringic acid	198.9
Gallic acid	168.9
Quercetin	301
p coumarci acid	162.9
Oleochantal	303.2
Hydroxytyrosol	153.05
Trans ferulic acid	193
Oleuropein	539
Hesperetin	301.3
Trimethoxyflavone	312
Arbutin	271.2
Rosmarinic acid	359
Ursolic acid	455
Apigenin	269
Amentoflavone	537.1
Luteoilin	284.9
Quercetin-3-O-glucoside	463.1
Quercetin-3-O- glucuronic acid	477
Kaempferol-3-O-glucose	609.1
Quercetin-3-O.hexose deoxyhexose	609.1
Isorhamnetin- 3-O Rutinoside	623.1
Isorhamnetin-7-O- Pentose	447.1
Luteoilin 7-O-glucoside	447.1
Kaempferol-3-O-glucuronic acid	461.1
Kaempferol-3-O-pentose	417.1
Kaempferol-3-O-hexose deohyhexose	593.1
Tyrosol	153.4
Protocathecoic acid	153
Vanillic acid	167
syringic acid	197
p-hydroxybenzoic\salicilic acid	137
Gentisic acid	153
Caffeic acid	179
Sinapic acid	223
Ferulic acid	193
Trans-cinnamic acid	147
Chlorogenic acid	353
Cathechin\epicathechin	289
Gallocathechin\epigallocathechin gallate	457
Gallocathechin\epigallocathechin	305
Cathechin gallate	441
Procianidin	577
Myricetin	317
Kaempeferol	285
Rutin	609
Narigin	579
Licopene	536.1
Delphinidin3-rutinoside-5-galactoside	772.8
Delphinidin-3-glucoside	465.2
Delphinidin-3-rutinoside	611
N-caffeoylputrescine	249
3-caffeoylquinic acid	353
Dihydroxycinnamoyl amide	470
N,N’-dicaffeoylspermidine	468
